# Introducing adjuvant-loaded particulate hepatitis B core antigen as an alternative therapeutic hepatitis B vaccine component

**DOI:** 10.1016/j.jhepr.2023.100997

**Published:** 2023-12-30

**Authors:** Jinpeng Su, Zahra Harati Taji, Anna D. Kosinska, Edanur Ates Oz, Zhe Xie, Pavlo Bielytskyi, Mikhail Shein, Philipp Hagen, Shohreh Esmaeili, Katja Steiger, Ulrike Protzer, Anne K. Schütz

**Affiliations:** 1 Institute of Virology, Technical University of Munich / Helmholtz Munich, 81675, Munich, Germany; 2 Ludwig Maximilians University of Munich, 81377, Munich, Germany; 3 Bavarian NMR Center, Technical University of Munich, 85748, Garching, Germany; 4 Institute of Structural Biology, Helmholtz Munich, 85764, Neuherberg, Germany; 5 German Center for Infection Research (DZIF), Munich partner site, Germany; 6 Comparative Experimental Pathology, Institute of Pathology, School of Medicine and Health, Technical University Munich, 81675, Munich, Germany

**Keywords:** particulate protein antigen, adjuvant packaging, chronic hepatitis B, therapeutic vaccine, *TherVacB*

## Abstract

**Background & Aims:**

Particulate hepatitis B core antigen (HBcoreAg) is a potent immunogen used as a vaccine carrier platform. HBcoreAg produced in *E. coli* encapsidates random bacterial RNA (*b*RNA). Using the heterologous protein-prime, viral-vector-boost therapeutic hepatitis B vaccine *TherVacB*, we compared the properties of different HBcoreAg forms. We explored how the content of HBcoreAg modulates antigen stability, immunogenicity, and antiviral efficacy.

**Methods:**

*b*RNA was removed from HBcoreAg by capsid disassembly, followed by reassembly in the absence or presence of specific nucleic acid-based adjuvants poly I:C or CpG. The morphology and structure of empty, *b*RNA-containing and adjuvant-loaded HBcoreAg were monitored by electron microscopy and nuclear magnetic resonance spectroscopy. Empty, *b*RNA-containing or adjuvant-loaded HBcoreAg were applied together with HBsAg and with or without nucleic acid-based external adjuvants within the *TherVacB* regimen in both wild-type and HBV-carrier mice.

**Results:**

While HBcoreAg retained its structure upon *b*RNA removal, its stability and immunogenicity decreased significantly. Loading HBcoreAg with nucleic acid-based adjuvants re-established stability of the capsid-like antigen. Immunization with poly I:C- or CpG-loaded HBcoreAg induced high antibody titers against co-administered HBsAg. When applied within the *TherVacB* regimen, they activated vigorous HBcoreAg- and HBsAg-specific T-cell responses in wild-type and HBV-carrier mice, requiring a significantly lower dose of adjuvant compared to externally added adjuvant. Finally, immunization with adjuvant-loaded HBcoreAg mixed with HBsAg led to long-term control of persistent HBV replication in the HBV-carrier mice.

**Conclusion:**

Adjuvant-loaded HBcoreAg retained capsid integrity and stability, was as immunogenic *in vivo* as externally adjuvanted HBcoreAg, requiring lower adjuvant levels, and supported immunity against co-administered, non-adjuvanted HBsAg. Thus, adjuvant-loaded HBcoreAg represents a promising novel platform for vaccine development.

**Impact and implications:**

Hepatitis B core antigen (HBcoreAg) recapitulates the capsid of the HBV that hosts the viral genome. Produced recombinantly, it is not infectious but emerges as a potent immunogen in vaccine development. In this preclinical study, we show that loading HBcoreAg with defined nucleic-acid-based adjuvants on the one hand stabilizes the HBcoreAg with standardized capsid content and, on the other hand, efficiently promotes the immunity of HBcoreAg and a co-administered antigen, allowing for reduced adjuvant doses. Therefore, adjuvant-loaded HBcoreAg not only serves as an encouraging option for therapeutic hepatitis B vaccines, but could also act as an efficient adjuvant delivery system for other types of vaccine.

## Highlights

• Recombinant particulate HBcoreAg, which is used for vaccine development, randomly packages bacterial RNA.
• Removal of bacterial RNA does not compromise particle integrity, but stability and immunogenicity decrease significantly.
• Loading HBcoreAg with defined nucleic acid-based adjuvants re-establishes the stability and immunogenicity of HBcoreAg.
• Adjuvant-loaded HBcoreAg strongly supports immune responses against co-administered antigens.
• Adjuvant-loaded HBcoreAg allows for a reduction of adjuvant dose, thereby reducing the risk of side effects.

## Graphical abstract

**Figure:**
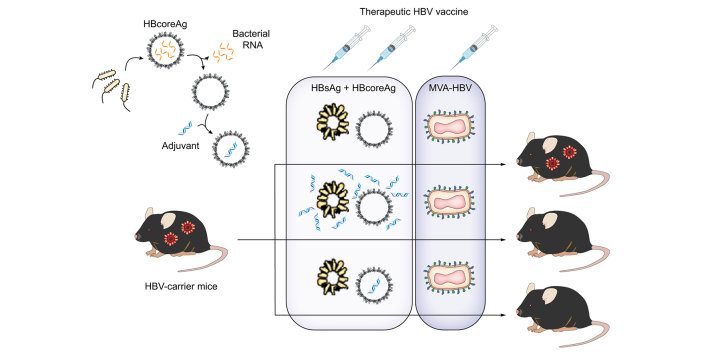

## Introduction

The first virus-like particle used as a vaccine carrier was based on the HBV capsid. This viral sub-structure, while not infectious on its own, is a highly immunogenic 36 nm particle that consists of 180 or 240 subunits of the HBV core protein forming homodimers and assembling spontaneously to an icosahedral particle referred to as hepatitis B core antigen (HBcoreAg). The self-assembly and high degree of immunogenicity are maintained when expressed as a recombinant protein. They can confer high immunogenicity on foreign antigens linked to the particle, either chemically or genetically.^1^

The HBV core protein consists of 183 amino acids that comprise the assembly (1-149) and the C-terminal nucleic acid binding (150-183) domains. The structure of HBcoreAg is characterized by a pattern of spikes on the surface of the capsid.^2^ The spikes comprise bundles of four α-helices contributed by two monomers. The large number of identical epitopes displayed as a regular pattern is likely one underlying determinant for HBcoreAg B-cell immunogenicity.^3^ In addition, immunodominant T-cell epitopes have been identified at the spike tip.^4^^,^^5^

In natural HBV infection, the viral capsid encloses the HBV pre-genomic RNA during assembly. Similarly, recombinant HBcoreAg encapsidates RNA, but in a non-specific manner.^6^ Therefore, HBcoreAg produced in *E. coli* self-assembles around heterologous bacterial RNA (*b*RNA). *b*RNA content in HBcoreAg produced in *E. coli* can vary between different batches due to the non-specific RNA encapsidation. HBcoreAg with encapsidated RNA has been proposed to promote T helper 1 (Th1) immunity.^7^^,^^8^ Reduction of the *b*RNA content by C-terminal truncation of the HBV core protein may lead to a switch from Th1 to Th2 responses.^9^

*b*RNA can be removed from HBcoreAg *in vitro* to obtain empty capsids^6^ or be replaced by specific nucleic acids of interest.^10^^,^^11^ Some nucleic acids are particularly known for their immunostimulatory effects and can be used as adjuvants to boost and shape the immune responses to a vaccine.^12^ One example is polyinosinic-polycytidylic acid (poly I:C), a synthetic analog of double-stranded RNA recognized by Toll-like receptor (TLR)3 and melanoma differentiation-associated protein-5 that enhances interferon (IFN) production, and induces a robust Th1 response.^12^ Another example is CpG oligonucleotides, short single-stranded DNA molecules containing unmethylated CpG dinucleotides, that trigger cells expressing TLR9 and promote the production of Th1-type and proinflammatory cytokines.^13^ Importantly, poly I:C and CpG adjuvants have been shown to enhance antigen-specific humoral responses and cellular responses^14^^,^^15^ and thus are exciting adjuvants, in particular for therapeutic vaccination.^16^

HBV infection is the leading cause of cirrhosis and hepatocellular carcinoma,^17^ which claims an estimated 880,000 lives annually and thus remains a major global health problem.^18^ Despite the availability of a safe and effective prophylactic vaccine, the WHO estimates that 296 million people worldwide suffer from chronic hepatitis B (CHB).^18^ The current standard treatment for CHB includes nucleotide analogs, which can efficiently suppress HBV replication but rarely cure the infection.^19^

While people with self-resolving acute HBV infection develop strong virus-specific CD4 and CD8 T-cell responses, patients with CHB develop a robust immune tolerance against HBV, characterized by scarce and partially dysfunctional virus-specific T cells and a lack of neutralizing antibodies. Thus, therapeutic vaccination that aims at restoring or inducing robust immune responses against HBV is a promising, potentially curative treatment for CHB.^20^

Vaccines containing only HBV surface antigen (HBsAg) could not achieve control of the virus in patients with CHB,^21^^,^^22^ indicating the need to induce a broader immune response to break HBV-specific immune tolerance. HBcoreAg-specific CD4 and CD8 T-cell responses are dominant in people with self-resolving infection or patients with CHB who can cure HBV.^21^ Still, such responses are almost absent in chronic HBV carriers.

We have, therefore, included both HBsAg and HBcoreAg as vaccine antigens in our previously developed therapeutic hepatitis B vaccine (*TherVacB*) that employs a heterologous prime-boost strategy.[23], [24], [25] Prime immunization with adjuvanted HBcoreAg and HBcoreAg induces neutralizing antibodies against HBsAg to prevent virus spread, activates CD4 T cells, and primes CD8 T cells against both antigens.^16^ Boost immunization with modified vaccinia virus Ankara (MVA) expressing HBV surface and core protein can then boost HBV-specific CD8 T cells to achieve control of the virus.^16^ In different preclinical mouse models of persistent HBV replication, *TherVacB* has been shown to attain long-term immune control of HBV by eliciting both HBsAg-specific and strong HBcoreAg-specific B- and T-cell responses.^26^ In our previous study, the success of *TherVacB* relied on incorporating a potent adjuvant to induce both Th1 and Th2 responses via prime immunization.^16^ Both poly I:C and CpG represent promising adjuvant options for the *TherVacB* regimen. Given the necessity of adjuvant inclusion in *TherVacB* and the encapsidation capability of HBcoreAg, we reasoned that HBcoreAg could be a suitable vehicle for the delivery of nucleic acid-based adjuvants, such as poly I:C and CpG. Such adjuvant-loaded HBcoreAg would be an exciting vaccine carrier and could improve the *TherVacB* regimen with fewer and better-controlled vaccine components. This study aimed to investigate the impact of the capsid content on the stability and immunogenicity of HBcoreAg using *TherVacB* as a prime example.

## Materials and methods

### HBcoreAg preparation

Recombinant *b*RNA HBcoreAg (genotype D, ayw) was expressed in *E. coli* BL21(DE3) and purified according to established protocols,^27^ followed by anion exchange chromatography using a HiTrap™ Q HP column (Cytvia Life Sciences, USA) to obtain HBcoreAg with uniform *b*RNA content. Empty HBcoreAg were prepared by removing *b*RNA through disassembling and reassembling HBcoreAg dimers.^6^^,^^27^

Adjuvant-loaded HBcoreAg was prepared by reassembling HBcoreAg dimers with 60-fold molar excess of poly I:C LMW (InvivoGen, USA) or 400-fold molar excess of CpG oligonucleotide 1668 (InvivoGen) by removing GnHCl using dilution or dialysis, respectively. Excess, unencapsidated adjuvant was removed using sucrose gradients.

### Transmission electron microscopy

Samples were prepared by adsorbing HBcoreAg on continuous carbon film supported copper grids (Plano, Wetzlar) followed by washing with 50 mM HEPES (pH 7.5) and negative staining with uranyl acetate solution (2% w/v). Images were acquired on a JEOL JEM-1400 Plus transmission electron microscope at 120 kV at 60,000x magnification (0.275 nm/pix) at 500 nm underfocus.

### Nuclear magnetic resonance spectroscopy

Solid-state nuclear magnetic resonance (NMR) spectroscopy was carried out on a 750 MHz Bruker AVANCE III NMR spectrometer with a 1.9-mm magic-angle spinning probe as described previously.^27^ ^13^C,^15^N-labelled *b*RNA or empty HBcoreAg were filled into a ZrO_2_ rotor (Bruker) and measured at a magic-angle spinning frequency of 16650 Hz with ^1^H and ^13^C 90° pulse lengths of 1.45 and 5.0 μs, respectively. The DARR spectrum was measured with a mixing time of 25 ms at a field of 16.65 kHz. Spectral processing and analysis were carried out with Bruker TopSpin 3.5 and CcpNmr Analysis,^28^ respectively.

The HBcoreAg was additionally evaluated using UV spectroscopy and ELISA. Detailed methods about the *in vitro* preparation and analysis can be found in the supplementary information.

### Ethical statement

Animal experiments were approved by the District Government of Upper Bavaria (permission number: ROB-55.2-2532.Vet_02-18-24). All experiments were carried out in strict compliance with the German regulations of the Society for Laboratory Animal Science (GV-SOLAS) and the European Health Law of the Federation of Laboratory Animal Science Associations (FELASA). The mice were maintained in specific pathogen-free, biosafety level 2 animal facilities in accordance with institutional guidelines. According to the regulations of the local animal welfare committee and the animal protection law, and to obey 3R principles, each experiment was performed once, including groups of 4-5 animals as indicated.

### Animal models

Wild-type, 8–10-week-old, C57BL/6J mice were purchased from Janvier Labs (Le Genest-Saint-Isle, France). In order to establish persistent HBV replication, wild-type C57BL/6J mice were intravenously injected with 4 × 10^9^ genome equivalents of adeno-associated virus (AAV)-HBV vector carrying a 1.2-fold overlength HBV genome (genotype D, ayw).^29^ AAV-HBV infected, HBV-carrier mice were bled shortly before initiating the immunization and grouped based on their serum levels of HBV e antigen (HBeAg) and HBsAg.

### Therapeutic hepatitis B vaccine regimen

Mice were immunized with a therapeutic, heterologous protein-prime/MVA-boost hepatitis B vaccine (*TherVacB*) as previously described.^23^ Briefly, mice were immunized intramuscularly twice with 10 μg of particulate HBsAg (genotype A, adw) and 10 μg of investigated HBcoreAg, followed by a boost with 3 × 10^7^ infectious units of recombinant MVA expressing HBV surface or core antigen, with a 2-week interval between each immunization. In the groups with external adjuvant, 30 μg of poly I:C LMW (InvivoGen, USA) or 10 μg of CpG oligonucleotide 1668 (InvivoGen) were formulated with HBsAg and empty or *b*RNA-containing HBcoreAg for the prime immunization.

### Serological analyses

Serum levels of HBsAg, HBeAg and anti-HBs were determined with the Architect™ platform (Abbott, Germany) as previously described.^26^ Serum levels of anti-HBc were quantified with the Liasion platform (DiaSorin, Italy). ELISA with anti-mouse IgG_1_ and IgG_2c_ antibodies were used to detect HBsAg (S-) and HBcoreAg (core)-specific IgG subclasses as described previously.^16^

### Detection of HBV-specific T cells by intracellular cytokine staining

Murine splenocytes and liver-associated lymphocytes were isolated and purified as previously described.^24^^,^^30^ For intracellular cytokine staining, cells were stimulated with 2 μg/ml of HBsAg or HBcoreAg-derived overlapping peptide pools^16^ for 16 h in the presence of 1 μg/ml of brefeldin A (Sigma-Aldrich, Germany). Ovalbumin-derived peptide OVA_S8L_ peptide (SIINFEKL) served as a control stimulant. Cell surface staining was carried out with anti-CD4 and anti-CD8 antibodies. Dead cells were excluded from analysis using the staining of Fixable Viability Dye eF780 (eBioscience, Germany). Intracellular IFNγ staining was carried out as previously described.^31^ The data were acquired on a CytoFlexS flow cytometer (Beckmann Coulter, USA) and analyzed with FlowJo software version 10 (Tree Star, USA).

### Immunohistochemistry

Murine liver tissues were fixed in paraformaldehyde for 48 h before being embedded in paraffin. Liver paraffin sections with 2 μm thickness were used to perform HBcoreAg-specific immunohistochemistry as previously described.^31^ Numbers of HBcoreAg-positive hepatocytes were determined from 10 random view fields under 20x magnification and quantified per mm^2^.

### Statistical analyses

Statistical analyses were performed in GraphPad Prism (GraphPad Software Inc., USA) using the Kruskal–Wallis test with Dunn’s multiple comparison correction, Mann-Whitney test, unpaired *t* test, and two-way ANOVA. *P* values <0.05 were considered significant. In experiments shown in Fig. 2, Fig. 4, Fig. 8, the non-vaccinated group had to be excluded from statistical analyses because identical values at the assay's baseline were detected in all mice.

## Results

### Empty HBcoreAg displays an identical structure to HBcoreAg containing *b*RNA

**Fig. 1:**
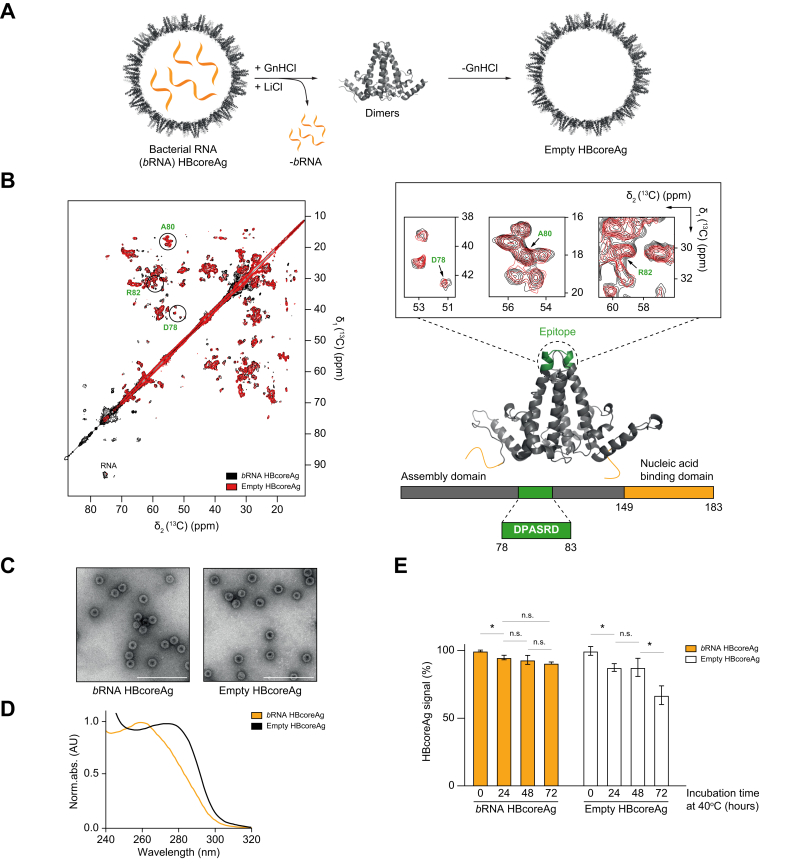
Preparation and characterization of empty and *b*RNA-containing HBcoreAg. (A) Scheme of empty HBcoreAg preparation. HBcoreAg was disassembled into dimers in the presence of GnHCl, and the released *b*RNA was removed by LiCl precipitation. The isolated HBcoreAg dimers were reassembled spontaneously upon GnHCl removal. (B) NMR spectra of ^13^C,^15^N-labelled *b*RNA-containing and empty HBcoreAg. The highlighted residues D78, A80, and R82 belong to the HBcoreAg epitope at the spike tip of the capsid. (C) Negative-stain transmission electron microscopy of *b*RNA and empty HBcoreAg. Scale bars indicate 200 nm. (D) UV spectroscopy of *b*RNA and empty HBcoreAg. (E) HBcoreAg-specific ELISA of *b*RNA and empty HBcoreAg. Samples for ELISA were incubated at 40 ^o^C for 0, 24, 48 or 72 h. *b*RNA and empty HBcoreAg without heat stress served as positive controls. Statistical analyses utilized the unpaired *t* test, ∗*p* <0.05. *b*RNA, bacterial RNA; GnHCl, guanidine HCl; HBcoreAg, HBV core antigen.

HBcoreAg produced in *E. coli* efficiently packages random *bRNA*, which results in heterogeneous capsid contents varying between different batches of HBcoreAg. To standardize capsid preparation, *b*RNA was removed from recombinantly produced HBcoreAg by disassembling the capsids (molecular weight five megadaltons) into their 120 constituent dimers (molecular weight 42 kilodaltons) in the presence of guanidine HCl (GnHCl). GnHCl disrupts electrostatic interactions between *b*RNA and the nucleic acid binding domain to support *b*RNA precipitation by LiCl. The dimers were purified, and GnHCl was removed to enable the reassembly of the dimers into empty HBcoreAg (Fig. 1A).

Solid-state NMR analyses showed that the empty and the *b*RNA-containing HBcoreAg displayed identical structural fingerprints at atomic resolution while the RNA signals disappeared in the empty HBcoreAg spectrum (Fig. 1B, left), in line with previous findings.^27^ This suggests that neither the disassembly and reassembly process nor the removal of nucleic acids impacted the structure of the assembly domain. Specifically, in the empty HBcoreAg spectrum, the immunodominant loop at the tips of the capsid spikes was not altered (Fig. 1B, right). Furthermore, the empty HBcoreAg retained the same morphology as *b*RNA HBcoreAg in negative-stain transmission electron microscopy (Fig. 1C). Thus, we ensured that any differences between the *b*RNA and empty HBcoreAg observed were due to the difference in capsid content but not structural changes in the core protein itself.

Throughout the preparation of empty HBcoreAg, UV absorption spectra were used to track the nucleic acid content. RNA removal was demonstrated by the shift in absorption maximum from 260 nm to 280 nm (Fig. 1D). While the isolated HBcoreAg dimers exhibited a 260 nm to 280 nm absorbance (260/280) ratio of 0.5-0.7 indicative of pure protein,^32^ the reassembled capsid has a higher 260/280 ratio (Fig. 1D), which is a result of light scattering due to the large size of the capsid.^6^

*b*RNA and empty HBcoreAg were subjected to ELISA analysis to confirm antigen integrity and immunogenicity^25^ before incorporation into the *TherVacB* regimen (Fig. 1E). However, it is known that empty HBcoreAg may be less stable due to a lack of electrostatic compensation for the highly negatively charged C-terminal domain.^6^ We therefore compared HBcoreAg stability following the WHO immunization management guidelines devised for the countries with logistical challenges, whereby vaccines are recommended to withstand a temperature of 40 °C for at least 3 days.^33^ *b*RNA and empty HBcoreAg were incubated at 40 °C for 0, 24, 48 or 72 h. *b*RNA HBcoreAg showed a minor 5% loss in ELISA signal after heating for 24 h, with no further significant loss observed with longer incubation. In contrast, empty HBcoreAg showed a gradual loss in ELISA signal over 72 h (Fig. 1E). Thus, empty HBcoreAg is less stable, although its structure is very comparable to *b*RNA HBcoreAg.

### Emptying HBcoreAg causes a decrease in its immunogenicity

**Fig. 2:**
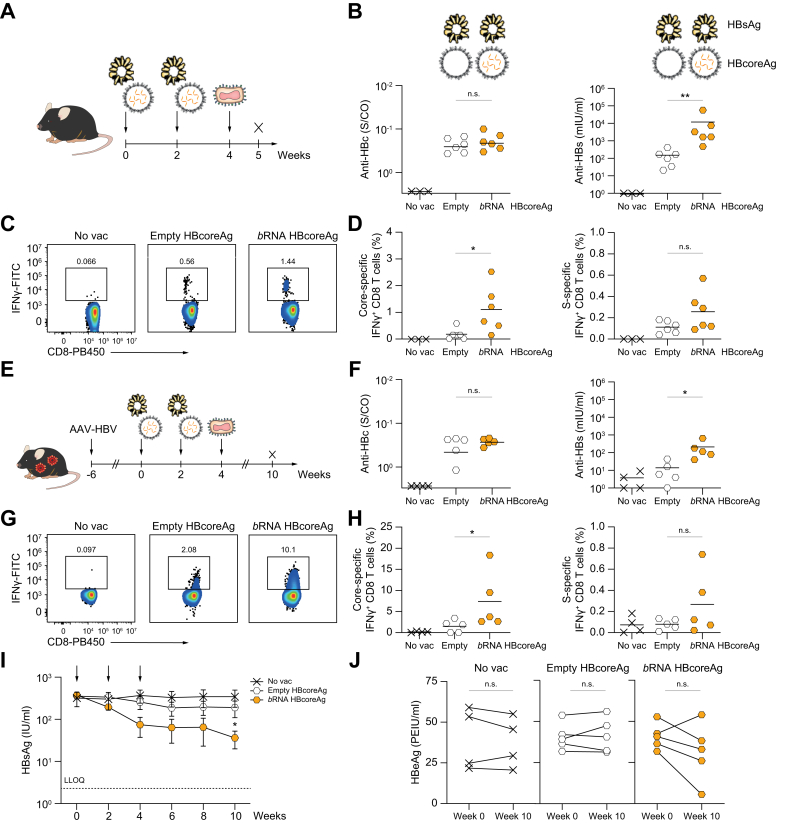
Immunogenicity of empty HBcoreAg in wild-type and HBV-carrier mice. (A) Wild-type C57BL/6J mice or (E) C57BL/6J mice infected with AAV-HBV 6 weeks before vaccination received two protein-prime vaccinations containing HBsAg and empty or *b*RNA-containing HBcoreAg at week 0 and 2, followed by an MVA-boost at week 4. Mice receiving no vaccination (no vac) served as controls. Endpoint analyses were performed at weeks 5 (B-D) and 10 (F-J). (B) Levels of serum anti-HBc (left) and anti-HBs (right). (C) Representative flow cytometry and (D) frequencies of splenic core-specific (left) or S-specific (right) IFNγ^+^ CD8 T cells determined by flow cytometry after intracellular cytokine staining following stimulation with a core- or S-specific peptide pool in wild-type mice. (F) Levels of serum anti-HBc (left) and anti-HBs (right). (G) Representative flow cytometry and (H) frequencies of intrahepatic core-specific (right) or S-specific (left) IFNγ^+^ CD8 T cells in HBV-carrier mice. (I) Time kinetics of serum HBsAg levels. Arrows indicate the vaccination time points. The dotted line indicates the LLOQ of the assay. An asterisk∗ indicates a significant difference to the no vac control. (J) Levels of serum HBeAg at week 0 and week 10. No vac group was excluded from statistical analyses because all values were equal to the assay baseline. Statistical analyses utilized the Mann-Whitney test or two-way ANOVA, ∗*p <*0.05, ∗∗*p <*0.01. AAV, adeno-associated virus; *b*RNA, bacterial RNA; HBcoreAg, HBV core antigen; HBeAg, HBV e antigen; HBsAg, HBV surface antigen; IFN, interferon; LLOQ, lower level of quantification.

In order to explore the impact of removing *b*RNA from HBcoreAg on its immunogenicity, we employed wild-type C57BL/6J mice or mice infected with an AAV vector transducing a replication-competent HBV genome that develop an HBV-carrier state. These wild-type (Fig. 2A-D) and HBV-carrier mice (Fig. 2E-J) were immunized with *TherVacB* utilizing empty and *b*RNA HBcoreAg. Specifically, mice were primed with particulate HBsAg together with either empty or *b*RNA HBcoreAg at week 0 and 2, and boosted with recombinant MVA expressing HBV surface and core proteins at week 4. Mice that did not receive any vaccination (no vac) served as controls. The experimental set-ups for wild-type and HBV-carrier mice are illustrated (Fig. 2A,E).

*TherVacB* immunization with empty and *b*RNA HBcoreAg induced comparably high levels of anti-HBc with respect to the non-vaccinated controls in both wild-type (Fig. 2B, left) and HBV-carrier mice (Fig. 2F, left). This is likely due to the large number of identical epitopes displayed on each capsid, which can directly activate B cells to produce anti-HBc as a T cell-independent antigen.^3^ By contrast, immunization with empty HBcoreAg elicited significantly lower splenic and intrahepatic core-specific CD8 T-cell responses compared to those in wild-type (Fig. 2C and left panel of 2D) and HBV-carrier mice (Fig. 2G and left panel of 2H) immunized with *b*RNA HBcoreAg, respectively. Very weak splenic CD8 T-cell responses were detected after immunization of both types of HBcoreAg in HBV-carrier mice, indicating that T cells were recruited to the liver where the antigen was expressed (Fig. S1).

Although all mice received the same amount of HBsAg, the S-specific antibody and CD8 T-cell responses in the empty HBcoreAg group were significantly lower than those in the *b*RNA HBcoreAg group. This indicates that HBcoreAg also supports HBsAg-specific immune responses and that a more immunogenic HBcoreAg does that more efficiently (right panels of Fig. 2B,D,F,H), as we have observed before.^25^

To evaluate the effect of the therapeutic vaccination on immune control of HBV, we monitored the serum HBsAg and HBeAg levels in HBV-carrier mice after *TherVacB* utilizing empty and *b*RNA HBcoreAg. While *TherVacB* utilizing *b*RNA HBcoreAg led to a significant HBsAg decrease, serum HBsAg in the empty HBcoreAg group remained unchanged and at similar levels to non-vaccinated controls (Fig. 2I). A drop of serum HBeAg was observed in four out of five mice that received *b*RNA HBcoreAg, but in none of the mice that received empty HBcoreAg or were not vaccinated (Fig. 2J). Taken together, the data demonstrates that the capsid content of HBcoreAg considerably influences the magnitudes of immune responses induced by HBcoreAg and co-administered HBsAg.

### HBcoreAg packages both DNA- and RNA-based adjuvants efficiently after *b*RNA removal

**Fig. 3:**
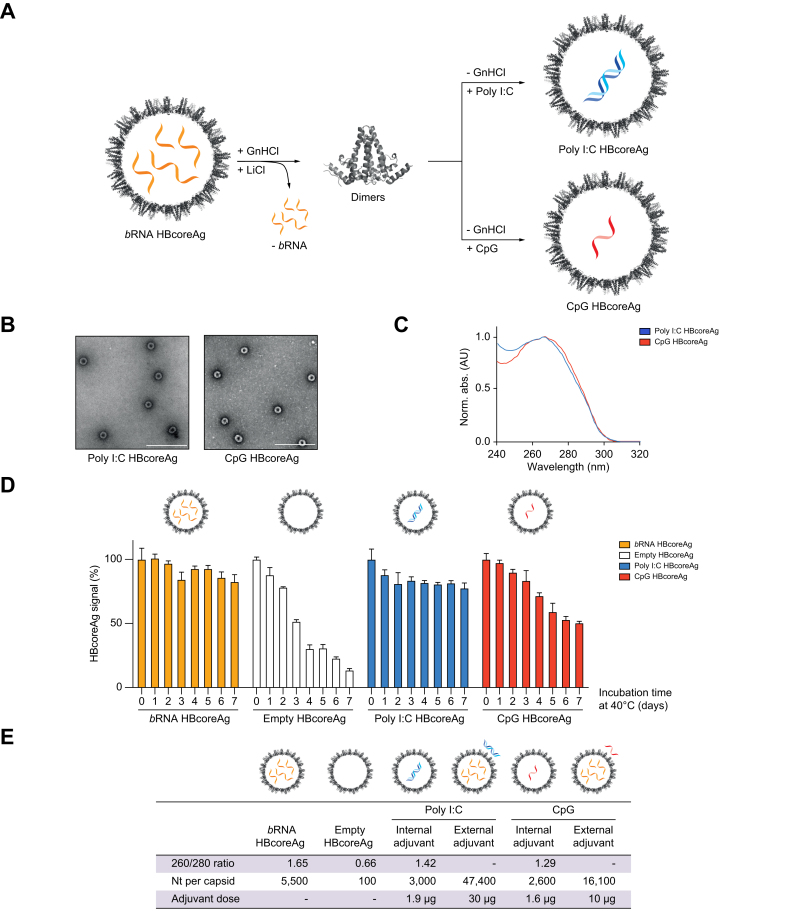
Preparation and characterization of adjuvant-loaded HBcoreAg. (A) Scheme of adjuvant-loaded HBcoreAg preparation. HBcoreAg dimers were isolated by disassembling *b*RNA HBcoreAg and removing *b*RNA; HBcoreAg was then allowed to re-assemble in the presence of the adjuvants, poly I:C or CpG. (B) Negative-stain transmission electron microscopy of poly I:C and CpG HBcoreAg. Scale bars indicate 200 nm. (C) UV spectroscopy of poly I:C and CpG HBcoreAg. (D) HBcoreAg-specific ELISA of *b*RNA, empty and adjuvant-loaded HBcoreAg. Samples were incubated at 40 °C for up to 7 days; HBcoreAg without heat stress served as a positive control. (E) The average number of encapsidated *vs*. external nucleotides per HBcoreAg group. The nucleotide content per capsid type was calculated from the UV absorption ratio at 260 nm to 280 nm. The adjuvant dose indicates the amount of adjuvant per 10 μg of HBcoreAg. *b*RNA, bacterial RNA; GnHCl, guanidine HCl; HBcoreAg, HBV core antigen; Poly I:C, polyinosinic-polycytidylic acid.

In order to take advantage of the favorable effects of nucleic acids on the immunogenicity of HBcoreAg, we substituted heterogenous *b*RNA by homogenous and well-characterized RNA-based poly I:C or DNA-based CpG to prepare adjuvant-loaded HBcoreAg for vaccine development. Poly I:C- and CpG-loaded HBcoreAg were prepared using a similar approach as used for empty HBcoreAg, where HBcoreAg dimers were disassembled and separated from *b*RNA and subsequently reassembled in the presence of either poly I:C or CpG (Fig. 3A). An excess of each adjuvant was used during capsid reassembly to ensure maximal adjuvant encapsidation, followed by sucrose gradient separation of adjuvant-loaded HBcoreAg from excess of free adjuvant. The negative-stain transmission electron microscopy revealed that both poly I:C and CpG HBcoreAg remained intact after adjuvant loading (Fig. 3B). The UV absorption maximum of adjuvant-loaded HBcoreAg shifted from 280 nm towards to 260 nm, similar to *b*RNA-containing HBcoreAg (Fig. 3C). Again, the thermostability of the HBcoreAg variants at 40 °C was evaluated over 7 days. While empty HBcoreAg rapidly lost their integrity, poly I:C was able to completely and CpG to partially compensate for the stability loss due to *b*RNA removal (Fig. 3D). Moreover, adjuvant-loaded HBcoreAg had a more homogeneous capsid content than *b*RNA HBcoreAg based on ion exchange chromatography (Fig. S2).

For the groups receiving externally adjuvanted protein antigens, HBcoreAg and HBsAg, the optimal dose for poly I:C was determined to be 30 μg based on the manufacturer’s recommendation and the results of a dose-titration study.^34^ For CpG adjuvant, a dose-titration study of CpG (Fig. S3) was performed, identifying 10 μg as the optimal dose for external adjuvant addition. The amount of adjuvant that may be encapsidated is limited by the capsid volume. The average adjuvant load per unit of HBcoreAg was calculated by quantification of the protein-to-RNA/DNA ratio (Fig. S4).^35^ Poly I:C HBcoreAg contained approximately 3,000 nucleotides per capsid, 16-fold lower than the amount of external poly I:C, while CpG HBcoreAg contained approximately 2,600 nucleotides per capsid, 6-fold less than the external CpG added to the proteins (Fig. 3E).

### Both poly I:C- and CpG-loaded HBcoreAg demonstrate strong immunogenicity in wild-type mice

Next, we studied whether the adjuvant-loaded capsids would suffice to activate immunity against HBcoreAg and co-administered HBsAg, and we compared these responses to those elicited by the externally adjuvanted mixture of HBsAg and HBcoreAg. The immunogenicity of adjuvant-loaded HBcoreAg was compared to that of externally adjuvanted empty or *b*RNA HBcoreAg mixed with HBsAg in wild-type C57BL/6J mice immunized following the *TherVacB* regimen.

**Fig. 4:**
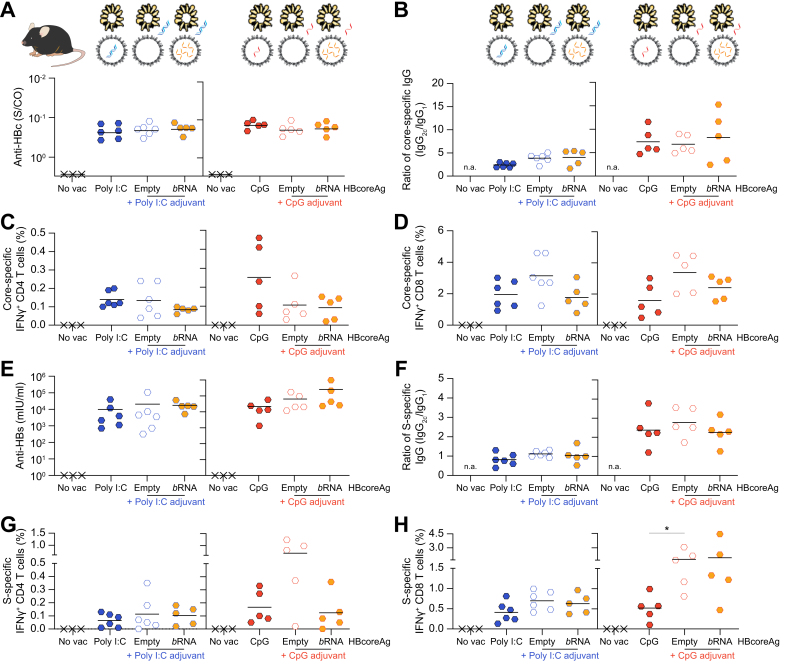
Immunogenicity of poly I:C and CpG HBcoreAg in wild-type mice. Wild-type C57BL/6J mice received two protein-prime vaccinations containing HBsAg together with empty/*b*RNA HBcoreAg supplemented with free poly I:C or CpG or with adjuvant-loaded HBcoreAg encapsidating the same adjuvant at week 0 and 2, followed by an MVA-boost at week 4. Mice receiving no vaccination (no vac) served as controls. Endpoint analyses were performed at week 5. (A-B) Levels (A) and IgG_2c_/IgG_1_ ratio (B) of serum anti-HBc in poly I:C-related (left) and CpG-related (right) groups. (C-D) Frequencies of splenic core-specific IFNγ^+^ CD4 (C) and CD8 (D) T cells determined by flow cytometry after intracellular cytokine staining following stimulation with a core-specific peptide pool in poly I:C-related (left) and CpG-related (right) groups. (E-F) Levels (E) and IgG_2c_/IgG_1_ ratio (F) of serum anti-HBs in poly I:C-related (left) and CpG-related (right) groups. (G-H) Frequencies of splenic S-specific IFNγ^+^ CD4 (G) and CD8 (H) T cells following stimulation with an S-specific peptide pool in poly I:C-related (left) and CpG-related (right) groups. n.a., not applicable. No vac group was excluded from statistical analyses because all mice had identical values (assay baseline). Statistical comparison utilized the Kruskal–Wallis test with Dunn’s multiple comparison correction, ∗*p <*0.05. Only statistically significant differences are indicated. *b*RNA, bacterial RNA; Core-, HBcoreAg; HBcoreAg, HBV core antigen; IFN, interferon; Poly I:C, polyinosinic-polycytidylic acid; S-, HBsAg.

*TherVacB* immunization with both poly I:C (Fig. 4A, left) and CpG HBcoreAg (Fig. 4A, right) induced comparably high levels of anti-HBc as observed in externally adjuvanted groups. Interestingly, the antibodies detected in all groups immunized with different types of HBcoreAg were predominantly Th1-type IgG_2c_ subclass (Fig. 4B), indicating that HBcoreAg promotes Th1-type immune responses irrespective of the capsid content. Intracellular IFNγ staining of splenocytes demonstrated that the frequencies of both core-specific IFNγ+ CD4 T cells (Fig. 4C) and CD8 T cells (Fig. 4D) were comparable between the mice immunized with poly I:C or CpG HBcoreAg and HBsAg and those vaccinated with an externally adjuvanted HBsAg and HBcoreAg mixture. Thus, both poly I:C and CpG HBcoreAg elicited comparable T-cell responses, although they contained significantly lower amounts of nucleic acids (Fig. 3E) compared to externally adjuvanted HBcoreAg.

Apart from strong core-specific immune responses, *TherVacB* immunization with poly I:C and CpG HBcoreAg activated serum anti-HBs titers as high as ≥10^4^ mIU/ml (Fig. 4E). Detection of S-specific IgG subclasses demonstrated that immunization with all vaccine formulations generated comparable levels of Th2-type IgG_1_ and Th1-type IgG_2c_ of anti-HBs (Fig. 4F), implying Th1/Th2-balanced S-specific responses induced by poly I:C and CpG adjuvants. Both poly I:C- and CpG-adjuvanted HBcoreAg elicited robust S-specific IFNγ+ CD4 T cells (Fig. 4G) and CD8 T cells (Fig. 4H, left), although the HBsAg was not formulated with adjuvant. Only the S-specific CD8 T-cell response induced by CpG HBcoreAg was lower than those in adjuvanted, empty, and *b*RNA HBcoreAg groups (Fig. 4H, right).

**Fig. 5:**
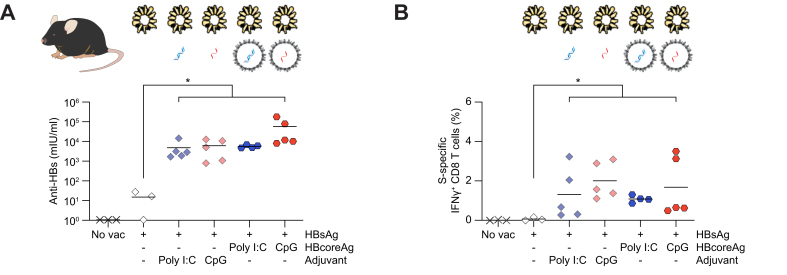
Effect of adjuvant-loaded HBcoreAg on co-administrated S-specific immune responses. C57BL/6J mice were immunized with HBsAg formulated either with free poly I:C or CpG adjuvant or with poly I:C- or CpG-loaded HBcoreAg at week 0 and 2, boosted with MVA at week 4 and analyzed at week 5. Non-vaccinated (no vac) mice served as controls. (A) Levels of serum anti-HBs. (B) Flow cytometry determined the frequency of splenic S-specific IFNγ+ CD8 T cells after ICS following stimulation with an S-specific peptide pool. HBcoreAg; HBcoreAg, HBV core antigen; IFN, interferon; MVA, modified vaccinia virus Ankara; S-, HBsAg; HBsAg, HBV surface antigen; Poly I:C, polyinosinic-polycytidylic acid.

A systematic comparison of different vaccine formulations revealed that combining HBsAg with either free poly I:C and CpG or poly I:C and CpG encapsidated into HBcoreAg significantly enhanced S-specific antibody and T-cell responses (Fig. 5). Hereby, the adjuvant formulation of HBsAg and the addition of poly I:C- and CpG-loaded HBcoreAg resulted in comparable levels of anti-HBs in serum (Fig. 5A) and S-specific CD8 T-cell responses in the liver (Fig. 5B), although the loaded HBcoreAg contains significantly lower amounts of nucleic acids. Taken together, lower adjuvant doses were sufficient to elicit robust B- and T-cell immunity when the adjuvant was encapsidated into HBcoreAg and applied together with HBsAg within the *TherVacB* regimen.

### *TherVacB* with both poly I:C- and CpG-loaded HBcoreAg leads to long-term control of persistent HBV replication in HBV-carrier mice

**Fig. 6:**
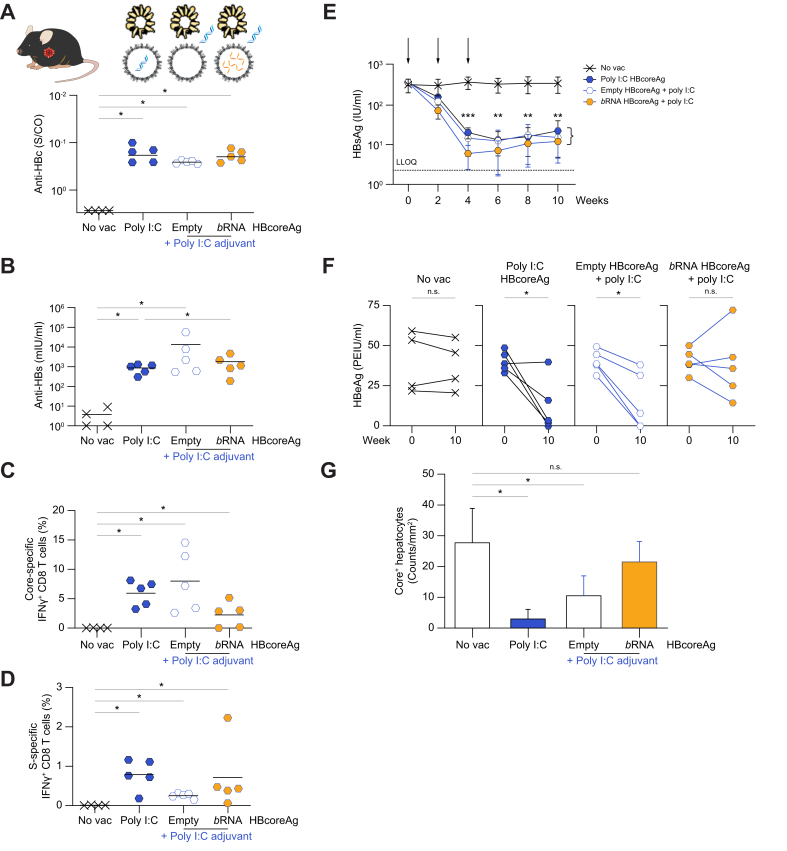
Immunogenicity comparison between poly I:C-loaded HBcoreAg and HBcoreAg adjuvanted with external poly I:C in HBV-carrier mice. AAV-HBV infected, HBV-carrier mice received two protein-prime vaccinations containing HBsAg together with poly I:C HBcoreAg or empty or *b*RNA-containing HBcoreAg with external poly I:C, followed by an MVA-boost in a 2-week interval. Non-vaccinated mice (no vac) served as controls. Endpoint analyses were performed at week 10. (A-B) Levels of serum anti-HBc (A) and anti-HBs (B). (C-D) Frequencies of intrahepatic core- (C) and S-specific (D) IFNγ+ CD8 T cells determined by flow cytometry after ICS following stimulation with the respective peptide pools. (E) Time kinetics of serum HBsAg levels. Arrows indicate vaccination time points. The dotted line indicates the LLOQ of the assay. An asterisk∗ indicates a significant difference to the no vac control. (F) Levels of serum HBeAg at week 0 and week 10. (G) Quantification of HBV core-positive hepatocytes detected by liver immunohistochemistry staining. Statistical analyses utilized the Kruskal–Wallis test with Dunn’s multiple comparison correction (A-D,G), two-way ANOVA (E), or Mann-Whitney test (F), ∗*p <*0.05, ∗∗*p <*0.01, ∗∗∗*p <*0.001. AAV, adeno-associated virus; *b*RNA, bacterial RNA; Core-, HBcoreAg; HBcoreAg, HBV core antigen; HBsAg, HBV surface antigen; IFN, interferon; LLOQ, lower level of quantification; MVA, modified vaccinia virus Ankara; Poly I:C, polyinosinic-polycytidylic acid; S-, HBsAg.

**Fig. 7:**
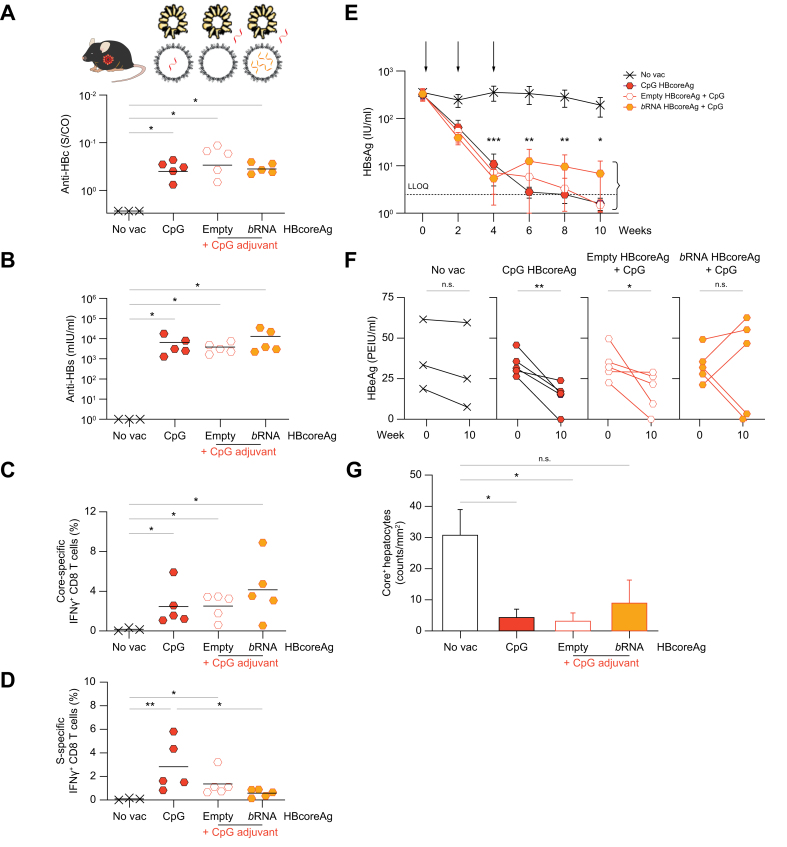
Immunogenicity comparison between CpG-loaded HBcoreAg and HBcoreAg adjuvanted with external CpG in HBV-carrier mice. AAV-HBV infected, HBV-carrier mice received two protein-prime vaccinations containing HBsAg together with CpG HBcoreAg or empty or *b*RNA HBcoreAg with external CpG, followed by an MVA-boost in a 2-week interval. Non-vaccinated mice (no vac) served as controls. Endpoint analyses were performed at week 10. (A-B) Levels of serum anti-HBc (A) and anti-HBs (B). (C-D) Frequencies of intrahepatic core- (C) and S-specific (D) IFNγ+ CD8 T cells determined by flow cytometry after intracellular cytokine staining following stimulation with the respective peptide pools. (E) Time kinetics of serum HBsAg levels. Arrows indicate the vaccination time points. The dotted line indicates the LLOQ of the assay. Asterisk∗ indicates the significance comparison to no vac control. (F) Levels of serum HBeAg at week 0 and week 10. (G) Quantification of HBV core-positive hepatocytes detected by liver immunohistochemistry staining. Statistical analyses using Kruskal–Wallis test with Dunn’s multiple comparison correction (A-D,G), two-way ANOVA (E), or Mann-Whitney test (F), ∗*p <*0.05, ∗∗*p <*0.01, ∗∗∗*p <*0.001. AAV, adeno-associated virus; *b*RNA, bacterial RNA; Core-, HBcoreAg; HBcoreAg, HBV core antigen; HBeAg, HBV e antigen; HBsAg, HBV surface antigen; IFN, interferon; LLOQ, lower level of quantification; MVA, modified vaccinia virus Ankara; S-, HBsAg.

To further explore the effects of immunization with the different HBcoreAg variants on antiviral immune control of HBV, the therapeutic effect of *TherVacB* using poly I:C-loaded (Fig. 6) and CpG-loaded HBcoreAg (Fig. 7) in comparison to externally adjuvanted protein antigens was evaluated in HBV-carrier mice that had developed a strong immune tolerance towards HBV.

In line with the observation in wild-type mice, vaccination of HBV-carrier mice with both poly I:C and CpG HBcoreAg induced strong anti-HBc (Fig. 6, Fig. 7A) and anti-HBs antibody responses (Fig. 6, Fig. 7B), and robust intrahepatic core-specific IFNγ+ CD8 T-cell responses (Fig. 6, Fig. 7C), similar to those using the externally adjuvanted protein antigens. Interestingly, and in contrast to what has been observed in wild-type mice (Fig. 4H), adjuvant-loaded HBcoreAg allowed for the induction of equal or even stronger intrahepatic S-specific IFNγ+ CD8 T-cell responses compared to externally adjuvanted protein antigens (Fig. 6, Fig. 7D), proving that *TherVacB* with adjuvant-loaded HBcoreAg can break immune tolerance against HBsAg and HBcoreAg in HBV-carrier mice.

To study the antiviral effect of the immunizations, we monitored the serum HBsAg and HBeAg levels and analyzed HBV core-positive hepatocytes in the livers of these mice. Immunization with both adjuvant-loaded and externally adjuvanted protein antigens resulted in a marked HBsAg decline using poly I:C HBcoreAg (Fig. 6E) and almost a complete loss of HBsAg using CpG HBcoreAg (Fig. 7E), whereas no decline was observed in non-vaccinated control mice. Furthermore, serum HBeAg significantly decreased after immunization with adjuvant-loaded HBcoreAg, but only in two mice when using external adjuvant and *b*RNA HBcoreAg (Fig. 6, Fig. 7F). In accordance with the decline of HBV antigen levels in serum, immunization with both poly I:C and CpG HBcoreAg led to a significant reduction of HBV core-positive hepatocytes at the end of the experiment (Fig. 6, Fig. 7G) proving that a curative antiviral immunity has been elicited in these groups.

Taken together, *TherVacB* vaccination utilizing both poly I:C- and CpG-loaded HBcoreAg is able to break HBV-specific immune tolerance, enabling the elimination of HBV-replicating hepatocytes and leading to long-term control of HBV in HBV-carrier mice.

### HBcoreAg loaded with poly I:C and CpG show comparable immunity and antiviral potency within *TherVacB* regimen

To investigate the influence of the type of capsid content on the immunogenicity of HBcoreAg, we compared the efficacy of poly I:C and CpG HBcoreAg in two parallel experiments in both wild-type and HBV-carrier mice, respectively. To avoid repeating *in vivo* experiments according to the 3R principle and to use as few animals as possible, the comparison was conducted parallel to the CpG HBcoreAg studies shown in Fig. 4, Fig. 7. Thus, the results of CpG HBcoreAg and no vac shown here are identical to those in the other two figures.

**Fig. 8:**
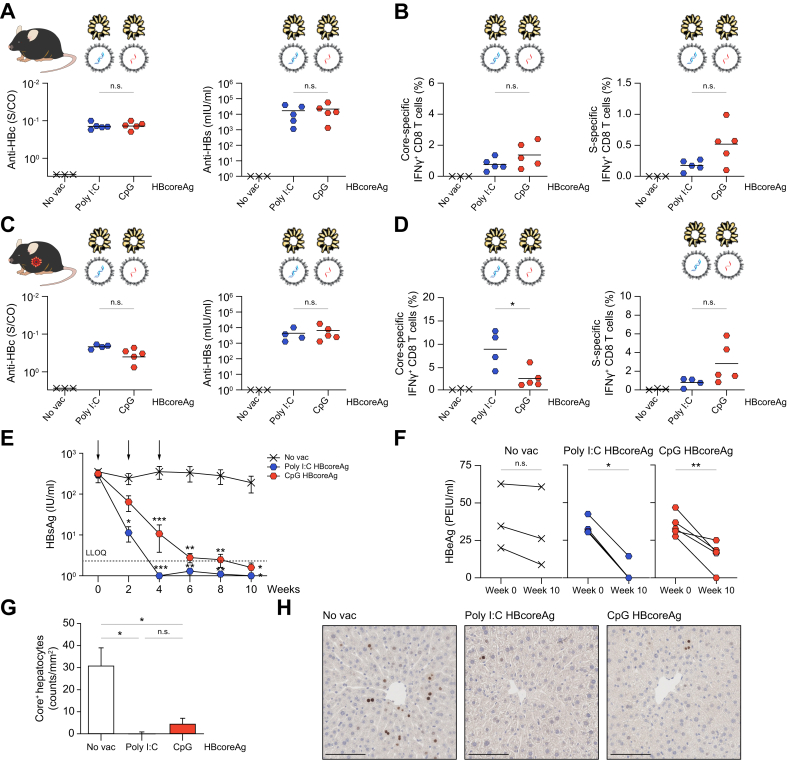
Immunogenicity comparison between CpG- and poly I:C-loaded HBcoreAg. (A-B) Wild-type C57BL/6J mice or (C–H) AAV-HBV infected, HBV-carrier mice were immunized with HBsAg together with poly I:C or CpG HBcoreAg at week 0 and 2, followed by an MVA-boost at week 4. Mice receiving no vaccination (no vac) served as controls. Endpoint analyses were performed at (A-B) week 5 and (C–F) week 10. The data for the CpG HBcoreAg group were extracted from Fig. 4, Fig. 6 above. (A, C) Levels of serum anti-HBc (left) and anti-HBs (right) in wild-type (A) and HBV-carrier (C) mice. (B, D) Frequencies of core-specific (left) and S-specific (right) IFNγ+ CD8 T cells determined by flow cytometry after intracellular cytokine staining following stimulation with respective peptide pools in the spleens of wild-type (B) and the livers of HBV-carrier (D) mice. (E) Time kinetics of serum HBsAg levels. Arrows indicate the vaccination time points. Dotted line indicates the LLOQ of the assay. Asterisk∗ indicates the significance comparison to no vac control. (F) Levels of serum HBeAg at week 0 and week 10. (G-H) Quantification (G) and representative images (H) of HBV core-positive hepatocytes (brown) detected by liver immunohistochemistry staining. Scale bars indicate 100 μm. No vac group was excluded from statistical analyses because of identical values (baseline of assay) in all mice. Statistical analyses utilized the Kruskal–Wallis test with Dunn’s multiple comparison correction (A-D,G), two-way ANOVA (E), or Mann-Whitney test (F), ∗*p <*0.05, ∗∗*p <*0.01, ∗∗∗*p <*0.001. AAV, adeno-associated virus; *b*RNA, bacterial RNA; Core-, HBcoreAg; HBcoreAg, HBV core antigen; HBeAg, HBV e antigen; HBsAg, HBV surface antigen; IFN, interferon; LLOQ, lower level of quantification; MVA, modified vaccinia virus Ankara; Poly I:C, polyinosinic-polycytidylic acid; S-, HBsAg.

In wild-type mice, *TherVacB* immunization with poly I:C and CpG HBcoreAg elicited comparably strong core- and S-specific antibodies (Fig. 8A) and CD8 T-cell responses (Fig. 8B) with a tendency towards stronger CD8 T-cell responses using CpG HBcoreAg. Similarly, the immunization with these two types of HBcoreAg led to equally high levels of anti-HBc and anti-HBs in HBV-carrier mice (Fig. 8C). However, immunization with poly I:C HBcoreAg induced more robust core-specific CD8 T-cell responses, whereas CpG HBcoreAg immunization resulted in more robust S-specific CD8 T-cell responses in HBV-carrier mice (Figs 8D and S5), indicating that antigen-specific immunity promoted by the two different adjuvants shows a distinct pattern, depending on which nucleic acid was encapsidated in the HBcoreAg.

The use of poly I:C HBcoreAg in *TherVacB* resulted in a rapid drop of HBsAg already after protein-prime vaccination, while the use of CpG HBcoreAg required the MVA-vector boost to control HBsAg in HBV-carrier mice (Fig. 8E). Three out of four HBV-carrier mice in the poly I:C HBcoreAg group but only one in four mice in the CpG HBcoreAg group lost HBeAg completely (Fig. 8F) and reduction of HBV core-positive hepatocytes showed a tendency to be stronger in the poly I:C HBcoreAg group (Fig. 8G,H). However, no statistically significant differences were observed between the poly I:C or CpG HBcoreAg conditions, and at the end of the experiments both approaches resulted in comparable antiviral effects. As these effects largely depend on the initial HBV antigen titers,^26^ smaller differences – like the ones observed – need to be interpreted with care.

Overall, both types of adjuvant-loaded HBcoreAg proved effective within the *TherVacB* regimen, breaking immune tolerance to HBcoreAg and co-administered HBsAg, and leading to immune control of persistent HBV infection through induction of a potent antiviral immune response.

## Discussion

HBcoreAg represents an interesting immunogen for vaccine development because it spontaneously forms regularly shaped particles. Recombinant HBcoreAg produced in *E. coli* packages irregular host *b*RNA, which may be unwanted for clinical development. Here, we evaluate the loading of HBcoreAg with nucleic acid-based adjuvants as an approach to stabilize the HBcoreAg and to promote immunity not only against HBcoreAg but also against the co-administered HBsAg.

We find that employing well-characterized nucleic acid-based adjuvants poly I:C or CpG to substitute *b*RNA could retain the capsid integrity and stability *in vitro* and significantly enhances the immunity of HBcoreAg *in vivo*. We demonstrate that removal of the randomly packed *b*RNA during HBcoreAg production in *E. coli* led to a loss of HBcoreAg stability and immunogenicity, whereas the substitution of *b*RNA with nucleic acid-based adjuvants poly I:C and CpG re-established both. *TherVacB* immunization with the adjuvant-loaded HBcoreAg induced strong HBV-specific humoral and cellular immune responses and led to sustained control of persistent HBV replication. Loading HBcoreAg with adjuvant provides an effective solution to stabilize the particulate antigen and allows the use of HBcoreAg as an efficient adjuvant delivery system.

Once regarded as "immunologists' dirty tricks",^36^ adjuvants have garnered intensive scrutiny regarding their safety. Numerous studies have reported an unwanted reactogenicity,^37^ local and systemic toxicity,^11^^,^^38^ and other adverse effects following adjuvant administration. Poly I:C and CpG, the adjuvants studied in this study, are no exception.^39^^,^^40^ Since it is mainly the adjuvant that triggers the reactogenicity of a vaccine in a dose-dependent manner, lowering the dose of adjuvant is an optimal way to achieve improved vaccine tolerability and safety.^41^ In our study, the adjuvant-loaded HBcoreAg contained about one order of magnitude less adjuvant than the minimum doses required to induce adequate immune responses by formulation with an external adjuvant. However, adjuvant-loaded HBcoreAg still achieved comparable or even stronger immune responses and antiviral effects than externally adjuvanted protein antigens. Most importantly, the adjuvant effect extended to the co-administered HBsAg. Therefore, utilizing adjuvant-loaded HBcoreAg presents a wise approach to decrease the adjuvant dose without compromising vaccine efficacy.

Both nucleic acids, poly I:C and CpG, have been broadly used as adjuvants in various vaccine settings. However, both of them are susceptible to nuclease degradation and exhibit a short half-life under physiological conditions.^42^^,^^43^ Our approach of encapsidating adjuvants in HBcoreAg represents an effective solution to improve adjuvant stability in the body by protecting them from various degradation pathways. In addition, it improves their targeted delivery to antigen-presenting cells, such as dendritic cells. Hereby, adjuvant-loaded HBcoreAg could work as a specific adjuvant delivery system for both HBcoreAg and co-administered antigens. Most likely, this is due to a process that has been described as intrastructural or intermolecular help.[44], [45], [46]

Adjuvant-loaded HBcoreAg elicited strong antiviral immunity when used within the *TherVacB* regimen, breaking the immune tolerance that had developed in HBV-carrier mice, and leading to significant and sustained antiviral effects. *TherVacB* vaccination with poly I:C-loaded HBcoreAg induced a more robust core-specific CD8 T-cell response, whereas CpG-loaded HBcoreAg stimulated a stronger S-specific CD8 T-cell response, at least in HBV-carrier mice. However, *TherVacB* immunization with either adjuvant-loaded HBcoreAg led to sustained control of persistent HBV replication.

Adding an external adjuvant to empty HBcoreAg elicited stronger immunity than adding it to *b*RNA-containing HBcoreAg. This could be explained by the formation of a physical complex between the nucleic acid binding domain of empty HBcoreAg and the external adjuvant that supports their co-delivery. In *b*RNA-containing HBcoreAg, the nucleic acid binding domain may be occupied by the encapsidated *b*RNA. It has been previously reported that the nucleic acid binding domain of the HBcoreAg can be exposed to the outside through the capsid pores when not interacting with RNA inside the capsid.^47^ Indeed, this exposure in the HBV life cycle allows for importin binding.^48^ We hypothesize that the nucleic acid binding domain of empty HBcoreAg might interact with external poly I:C and CpG to assist with site-specific delivery of the adjuvant (Fig. S6).

It should be noted, however, that empty HBcoreAg is significantly less stable than its nucleic acid-containing counterparts. Thus, the preferred choice is to employ nucleic acid-containing HBcoreAg in the *TherVacB* regimen. The major drawback of utilizing adjuvant-loaded HBcoreAg is the complicated production procedure with resulting low yields and high costs. Nevertheless, as a proof of concept, our work demonstrates that adjuvant-loaded HBcoreAg is a tool to efficiently deliver adjuvants, enhancing the immunity of HBcoreAg itself and of a co-administered antigen.

In summary, our findings establish that the HBcoreAg content critically determines its stability and immunogenicity. HBcoreAg loaded with the nucleic acid-based adjuvants poly I:C and CpG induced strong immune responses against HBcoreAg and against the co-administered HBsAg and led to long-term control of persistent HBV infection when applied within the *TherVacB* regimen. Adjuvant-loaded HBcoreAg provides a targeted and efficient mechanism of adjuvant delivery, allowing for a reduction of the adjuvant dose to avoid potential side effects without compromising immunostimulatory effects. Thus, adjuvant-loaded HBcoreAg represents a promising platform for therapeutic HBV vaccines and other vaccine regimens that can be enhanced through the targeted delivery of adjuvants in controlled doses.

## Financial support

This project received funding from the German Research Foundation (DFG) via SFB-TRR 179/2 (2020–272983813, project 18 to UP and 23 to AKS) and the Emmy Noether program (2018–394455587 to AKS), from Horizon 2020 - European Commission via the TherVacB consortium (grant agreement No. 848223, to UP) and by the German Ministry of Education and Research (BMBF) via project TherVacB PLUS.

## Authors’ contributions

JSu, ZHT, AKS, UP designed the study; JSu, ADK, EAO, ZX, PH performed *in vivo* experiments; ZHT, PB, MS, SE performed *in vitro* experiments; KS supported the immunohistochemistry analysis of mouse liver tissues; AKS and UP provided supervision and funding; JSu, ZHT, AKS, UP wrote and finalized the manuscript. All authors read and approved the final version of the manuscript.

## Data availability statement

All source data that support the findings of this study are available from the corresponding authors, AKS (in vitro part) and UP (in vivo part), upon reasonable request.

## Conflict of interest

UP is a co-founder, shareholder, and board member of SCG Cell Therapy. UP serves as ad hoc advisor for Abbott, Aligos, Arbutus, Gilead, GSK, Merck, Sanofi, Roche, and VirBiotech. UP and ADK are named as inventors on a patent application describing the therapeutic vaccination scheme *TherVacB* (PCT/EP2017/050553). The remaining authors declare no competing interests.

Please refer to the accompanying ICMJE disclosure forms for further details.
