# Supplementary material for: Introducing adjuvant-loaded particulate hepatitis B core antigen as an alternative therapeutic hepatitis B vaccine component
Source: JHEP Rep. 2023 Dec 30;6(4):100997. doi: 10.1016/j.jhepr.2023.100997 (PMC10904195; doi:10.1016/j.jhepr.2023.100997)
Supplement: Multimedia component 1 [file mmc1.pdf]

Introducing adjuvant-loaded particulate hepatitis B core antigen as an alternative therapeutic hepatitis B vaccine component

Jinpeng Su, Zahra Harati Taji, Anna D. Kosinska, Edanur Ates Oz, Zhe Xie, Pavlo Bielytskyi, Mikhail Shein, Philipp Hagen, Shohreh Esmaeili, Katja Steiger, Ulrike Protzer, Anne K. Schütz

Table of contents

Supplementary Methods 2

Fig. S1. 7

Fig. S2. 8

Fig. S3 9

Fig. S4. 11

Fig. S5. 13

Fig. S6. 15

Supplementary References 16

Supplementary Methods

HBcoreAg Preparation

Recombinant HBcoreAg (genotype D, ayw) containing bacterial RNA (*b*RNA) were produced using a pRSF1b-based vector expressed in *E. coli* BL21(DE3) cells. The *b*RNA-containing HBcoreAg were purified according to established protocols [1]. Briefly, harvested cells were lysed by sonication in a TRIS-based buffer with 5 mM EDTA, 10 mM $MgCl_2$ and 0.2% Igepal CA-650 supplemented with DNAse I and protease inhibitor (SERVA, Heidelberg). The lysate was spun down and the filtered supernatant was loaded onto a sucrose gradient (10% to 60% sucrose). The gradient was spun in an ultracentrifuge (134,000×g, 4°C, 15 hours). The gradients were delayered and the HBcoreAg-containing fractions were identified using SDS-PAGE. The combined capsid fractions were precipitated using $(NH_4)_2SO_4$ to 60 % saturation. The precipitated capsids were spun down, resuspended and dialyzed overnight at 4°C into PBS. The HBcoreAg were further run over a HiTrap™ Q HP anion exchange chromatography column (Cytvia Life Sciences, USA) and the fractions of the main capsid peak with uniform RNA content (using UV absorption ratios A_{260}/A_{280}) were combined. *b*RNA HBcoreAg were concentrated using 100 kDa molecular weight cut-off (MWCO) centrifugal filtration units (Merck Millipore, USA), supplemented with 5% glycerol, sterile-filtered and flash-frozen with liquid N_2. The HBcoreAg was stored at -80°C until needed and thawed at 4°C. Protein concentration was determined using a Bradford assay and capsid integrity was controlled using HBcoreAg-specific ELISA and TEM.

Preparation of empty HBcoreAg

Purified *b*RNA-containing HBcoreAg were emptied of unspecific *b*RNA as previously reported [1, 2]. Briefly, HBcoreAg were disassembled overnight in 50 mM HEPES (pH 7.5), 1.5 M GnHCl, 0.5 M LiCl (Sigma Aldrich, USA), 2 mM DTT overnight. The bacterial RNA precipitated with LiCl was removed by centrifugation and the dimers were purified on a Superdex 200 column (Cytvia Life Sciences). The isolated dimers were reassembled by dialysis into 50 mM HEPES, 250 mM NaCl, 2 mM DTT overnight. The empty HBcoreAg were then dialyzed into PBS, concentrated using 100 kDa (MWCO) centrifugal filtration units (Merck Millipore), supplemented with 5% glycerol, sterile-filtered and flash-frozen with liquid N_2. The HBcoreAg were stored at -80°C until needed and thawed at 4°C. Protein concentration was determined using a Bradford assay and HBcoreAg integrity was controlled using HBcoreAg-specific ELISA and TEM.

Preparation of poly I:C and CpG HBcoreAg

For adjuvant-loaded HBcoreAg, HBcoreAg dimers were purified as described above, concentrated using 30 kDa MWCO centrifugal filtration units (Merck Millipore), then reassembled in the presence of poly I:C LMW (InvivoGen, USA) or CpG oligonucleotide 1668 (InvivoGen). Poly I:C LMW is a double-stranded RNA with an average size of 200 to 1000 kb. CpG ODN 1668 is a single-stranded 20-mer DNA with phosphorothioate-linked bases. For poly I:C HBcoreAg, the dimers were diluted 1 in 3 (to a final GnHCl concentration of 0.5 M) with the addition of 60-fold molar excess poly I:C. They were then allowed to stir at room temperature for 30 minutes. For CpG

HBcoreAg, the dimers were added to a dialysis cassette (Thermo Fisher Scientific, USA) and dialyzed against 50 mM HEPES, 350 mM NaCl, 2 mM DTT at 4°C while 400-fold molar excess CpG was added dropwise to the cassette. The CpG encapsidation was carried out more slowly and at lower temperature than poly I:C encapsidation, as CpG HBcoreAg incorporate less nucleic acids than poly I:C HBcoreAg and should therefore be handled more gently. The dialysis was continued at 4°C for 4 hours. In order to remove excess, unencapsidated RNA and DNA, both poly I:C and CpG HBcoreAg were loaded onto sucrose gradients (10% to 60% sucrose). The gradients were spun in an ultracentrifuge (134,000×g, 4°C, 15 hours). After delayering, the fractions containing free poly I:C or CpG were identified using Native Agarose Gel Electrophoresis while the fractions containing poly I:C or CpG HBcoreAg were identified using SDS-PAGE. The poly I:C and CpG HBcoreAg were then dialyzed into PBS, concentrated using 100 kDa MWCO centrifugal filtration units (Merck Millipore), supplemented with 5% glycerol, sterile-filtered and flash-frozen with liquid N_2. The HBcoreAg were stored at -80°C until needed and thawed at 4°C. Protein concentration was determined using a Bradford assay and HBcoreAg integrity was controlled using HBcoreAg-specific ELISA and TEM.

Sample storage

*b*RNA, empty, poly I:C and CpG HBcoreAg dialyzed into PBS and supplemented with 5% glycerol were sterile filtered, flash-frozen in liquid N_2 and stored at -80°C until use.

Transmission Electron Microscopy

Samples for TEM were prepared on continuous carbon film supported copper grids (Plano, Wetzlar). After glow discharge sputtering, 5 µL of HBcoreAg was added to the grid, incubated for 5 minutes and removed by washing with 20 µL of 50 mM HEPES (pH 7.5) buffer. The grid was stained with 5 µL uranyl acetate solution (2 % w/v) for 30 seconds. Upon removal of the staining solution, the grid was air-dried and stored at room temperature until imaged. Images were acquired on a JEOL JEM-1400 Plus transmission electron microscope at 120 kV, with a charge-coupled device camera (JEOL CCD Ruby, 8 Mpix), at 60,000x magnification (0.275 nm/pix) at 500 nm underfocus.

Nuclear magnetic resonance spectroscopy

HBcoreAg solid-state NMR spectroscopy measurements were performed on a 750 MHz Bruker AVANCE III NMR spectrometer with a 1.9-mm Magic-Angle Spinning (MAS) probe, as previously described [1]. Briefly, 9 mg of ^{13}C,^{15}N-labelled *b*RNA or empty HBcoreAg were filled into a ZrO_2 rotor (Bruker, USA). Experiments were conducted at a MAS frequency of 16650 Hz with ^{1}H and ^{13}C 90° pulse lengths of 1.45 and 5.0 µs, respectively. The DARR spectrum was measured with a mixing time of 25 ms at a field of 16.65 kHz. The sample temperature was 14°C and the spectrum was referenced to trimethylsilylpropanesulfonate (Sigma Aldrich, USA). Spectral processing and analysis were carried out with Bruker TopSpin 3.5 and CcpNmr Analysis [3], respectively.

UV spectroscopy

UV spectra were acquired on an Implen NanoPhotometer N60 with background subtraction. The relative protein and nucleic acid concentrations of poly I:C and CpG HBcoreAg were determined according to Figure S1.

ELISA of different types of HBcoreAg

HBcoreAg-specific sandwich ELISA was performed as previously described [4]. For the initial comparison of *b*RNA and empty HBcoreAg, wells were coated with anti-HBcoreAg 17H7 antibody (1 µg/mL, 100 µL/well) kindly provided by the Helmholtz Monoclonal Antibody Core Facility, and detected with HRP-labeled secondary antibody (1:45000) (Sigma Aldrich, USA). 5 µg of HBcoreAg was used for analysis. For the extended 7-day stability series, wells were coated with anti IFA HepBCore (1 µg/mL, 100 µL/well) and detected with HRP-labeled secondary antibody (1:7000) (Centro de Ingeniería Genética y Biotecnología de Cuba). 80 ng of HBcoreAg was used for analysis. For stability screening, samples were incubated at 40°C for up to 7 days prior to ELISA and compared to the respective HBcoreAg variants without heat stress.

Supplementary Figures

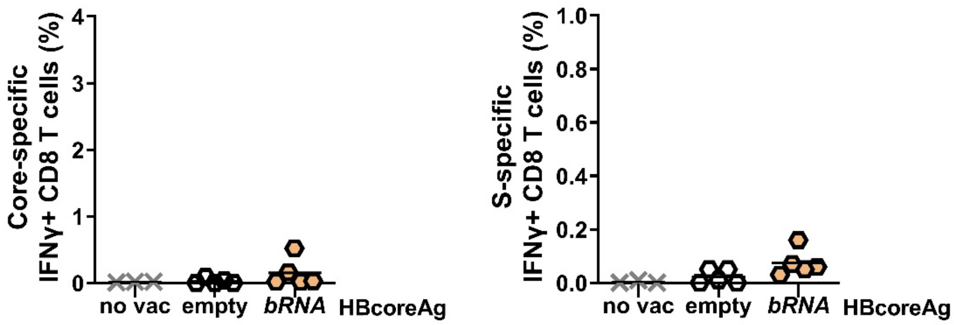

Fig. S1: HBV-specific immune responses in the spleen of HBV-carrier mice after TherVacB immunization with *b*RNA or empty HBcoreAg.

C57BL/6J mice infected with AAV-HBV six weeks prior to vaccination received two protein-prime vaccinations containing HBsAg and empty or *b*RNA HBcoreAg at week 0 and 2, followed by an MVA-boost at week 4. Mice receiving no vaccination (no vac) served as controls. Endpoint analyses were performed at week 10. Frequencies of splenic core-specific (left) or S-specific (right) IFNγ^{+} CD8 T cells determined by flow cytometry after intracellular cytokine staining (ICS) following stimulation with a core- or S-specific peptide pool in HBV-carrier mice.

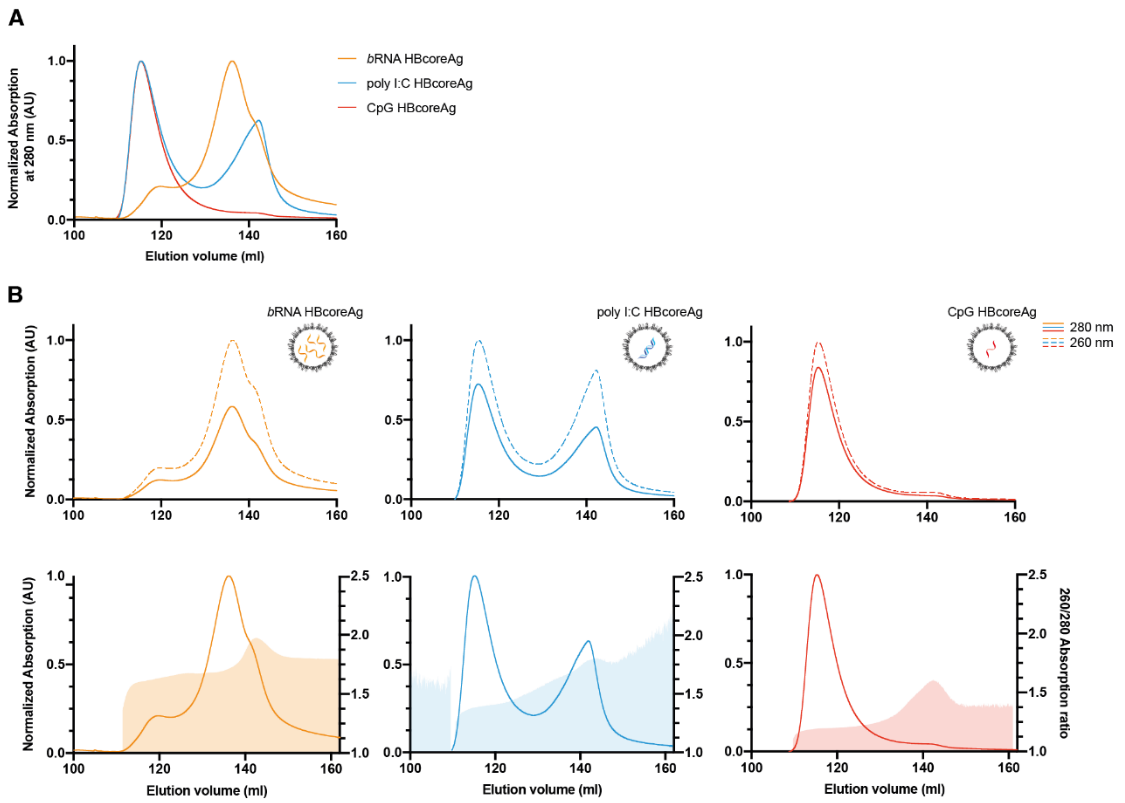

Fig. S2: Ion exchange chromatography of *b*RNA-containing, poly I:C- and CpG-loaded HBcoreAg.

*b*RNA-containing, poly- I:C and CpG-loaded HBcoreAg were run over HiTrap Q HP (Cytiva Life Sciences, USA) anion exchange chromatography column after final purification, with a linear gradient of 20-100% 1M NaCl in 10 mM MES (pH 6) buffer. (A) Overlay of ion exchange chromatograms from *b*RNA-containing, poly I:C- and CpG-loaded HBcoreAg with normalized absorption at 280 nm. (B) Individual chromatograms with 260 and 280 nm absorption traces for *b*RNA-containing (left), poly I:C- (center) and CpG-loaded (right) HBcoreAg. The highlighted area tracks the change in 260 to 280 nm absorption ratio to determine the nucleotide content of the capsid. The traces represent the distribution of charges within a batch of capsids, which can reflect the number of enclosed nucleotides as it tracks with the varying 260 to 280 nm absorption ratio. *b*RNA HBcoreAg show a broader, continuous distribution of varying capsid fillings compared to the adjuvant-loaded HBcoreAg.

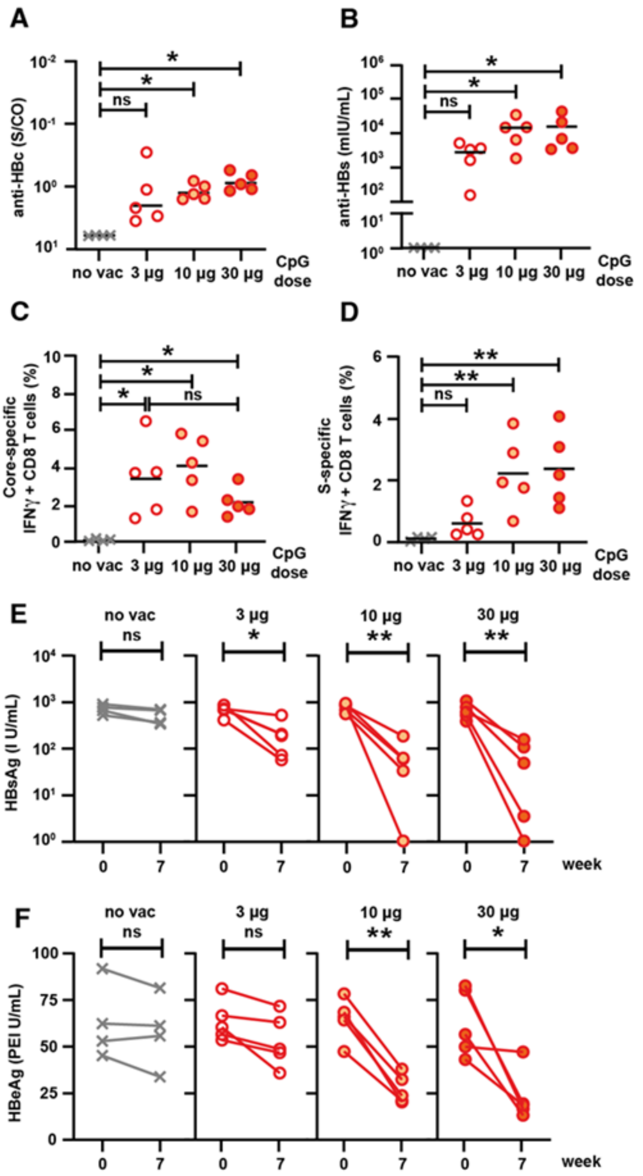

Fig. S3. Immune responses induced by *TherVacB* immunization with different doses of CpG adjuvant.

C57BL/6J mice were intravenously injected with AAV-HBV vector six weeks prior to vaccination to establish persistent HBV infection. Afterwards, mice were immunized with HBsAg/HBcoreAg (APP Lativijas Biomedicinas, Latvia) together with 3µg, 10µg, or 30µg CpG at week 0 and 2, followed by an MVA-boost at week 4. Non-vaccinated mice (no vac) served as controls. The final analyses were performed at week 7. (A-B) Levels of serum anti-HBc (A) and anti-HBs (B). (C-D) Frequencies of intrahepatic core-specific (C) and S-specific (D) IFNγ+ CD8 T cells determined by flow cytometry

after ICS following stimulation with the respective peptide pools. (E-F) Levels of serum HBsAg (E) and HBeAg (F) at week 0 (start point) and week 7 (endpoint).

Adjuvant encapsidated in HBcoreAg	poly I:C HBcoreAg	CpG HBcoreAg
Protein extinction coefficient at 260 nm, ε_{260}	18,300	
Protein extinction coefficient at 280 nm, ε_{280}	30,500	
RNA or DNA extinction coefficient at 260 nm, ε_{260}	7,400	9,800
RNA or DNA extinction coefficient at 280 nm, ε_{280}	3,700	6,100
Molar ratio of nucleotides to protein monomer	12.5	10.8
Number of nucleotides per capsid	3,000	2,600
Average molecular weight per nucleotide (Da)	317	310
Molecular weight of nucleotides per capsid (kDa)	951	806
Capsid molecular weight (kDa)	5,000	
Mass of RNA or DNA per 10 µg of capsid (µg)	1.9	1.6

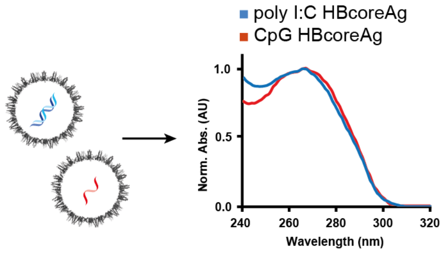

Externally administered adjuvant	External poly I:C	External CpG
Mass of RNA or DNA per 10 µg of capsid (µg)	30	10
Average molecular weight per nucleotide (Da)	317	310
Moles of nucleotides per 10 µg of capsid (pmol)	94,600	32,300
Capsid molecular weight (kDa)	5,000	
Moles of capsid per 10 µg (pmol)	2	
Number of nucleotides per capsid	47,300	16,150

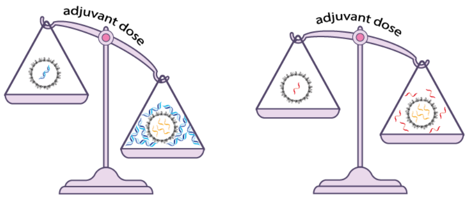

Fig. S4. Comparison of adjuvant dose in adjuvant-loaded and externally adjuvanted HBcoreAg groups.

UV spectra of adjuvant-loaded HBcoreAg were measured on Implen NanoPhotometer N60 with background subtraction. The absorption values at 260 and 280 nm were corrected for light scattering using absorption values at 340 and 360 nm, then used to determine protein and nucleic acid concentrations using the extinction coefficients at 260 (ε_{260}) and 280 (ε_{280}) nm [5]. The ε_{260} and ε_{280} for HBcoreAg have been previously reported [5]. For the adjuvants, the ε_{260} was calculated using the ε_{260} of the individual nucleotides [5-7]. The ε_{280} was then determined using the reported $\varepsilon_{260}/\varepsilon_{280}$ ratio of 2 and 1.6 for pure RNA (for poly I:C) and DNA (for CpG), respectively [5, 8]. The $\varepsilon_{260}/\varepsilon_{280}$ ratio of pure DNA is often reported to be as high as 1.8 [5], which would reduce the estimated number of nucleotides per capsid even further to 1,800. The ratio of nucleotides to protein monomer were used to determine the number of nucleotides per capsid. The number of nucleotides per capsid were about 15% higher in poly I:C HBcoreAg than CpG HBcoreAg, leading to enhanced charge compensation between the positively charged C-terminal domain and the negatively charged nucleotides. This

compensation contributes to the better heat stability observed for poly I:C HBcoreAg (Figure 3D). For externally adjuvanted groups, the adjuvant doses were chosen based on *in vivo* studies (Figure S3, [9]), as outlined above. Average molecular weight per nucleotide is provided as the average for the respective RNA or DNA molecule.

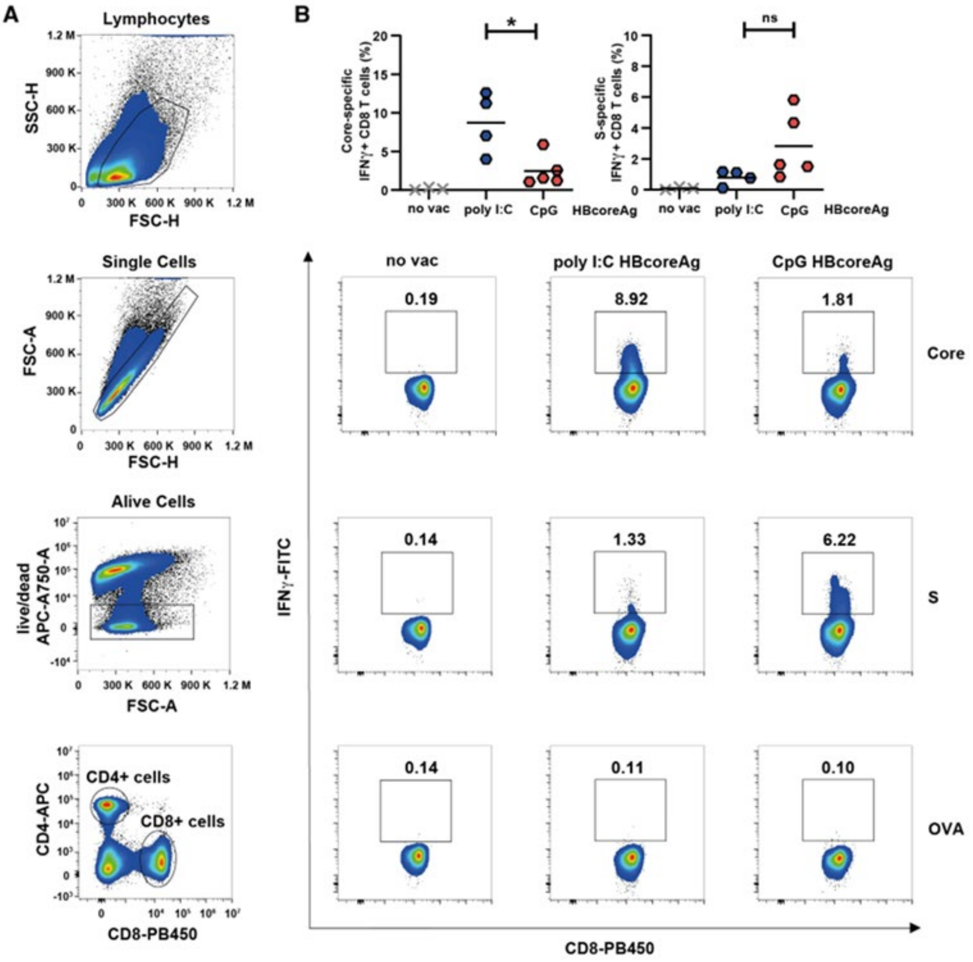

Supplement Fig. S5. Gating strategy and representative plots of flow cytometric analysis.

(A) Gating strategy of flow cytometric analysis. The forward- vs sides-scattered-light plot was used to exclude cell debris and gate lymphocyte population (1st diagram, vertically). Single cells were selected from lymphocyte population by plotting height vs area of forward-scattered-light (FSC-H vs FSC-A) and gating on the diagonal population (2nd diagram, vertically). Alive cells from single cell population were defined by exclusion of the fixable viability dye (FVD)-APC-A750-positive cells (3rd diagram, vertically). CD4 or CD8 T cells were gated by plotting CD4-APC against CD8-PB450 within viable lymphocyte population (4th diagram, vertically). (B) Frequencies of core- and S-specific IFNγ+ CD8 T cells (upper panel) and representative flow cytometry stainings from one representative mouse (lower panel). IFNγ+ CD8 T cells were defined by gating IFNγ-FITC against CD8-PB450. The control stimulated with

ovalbumin (OVA)-derived peptide was used to set up the gate position. APC, allophycocyanin; PB450, Pacific Blue 450; FITC, fluorescein isothiocyanate.

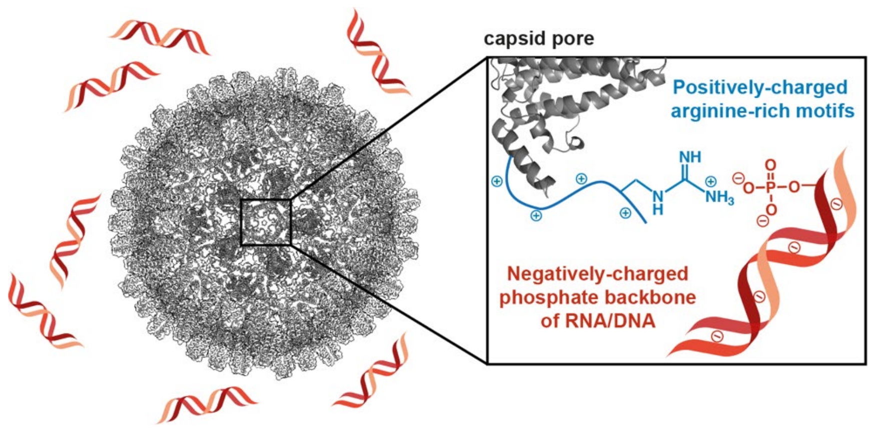

Fig. S6. Model for the interaction of external adjuvant with empty HBcoreAg.

The nucleic acid binding domain of HBcoreAg contains a series of arginine-rich motifs, leading to a high concentration of positive charges at the flexible C-terminus. In empty HBcoreAg, the nucleic acid binding domain can protrude out of the capsid pores to potentially interact with the negatively-charged phosphate backbone of an RNA- or DNA-based adjuvant. We hypothesize that this interaction could favor a co-delivery of external adjuvant with empty HBcoreAg, which could explain the stronger antiviral effect compared to externally adjuvanted *b*RNA HBcoreAg.

Supplementary References

Author names in bold designate shared co-first authorship

[1] **Harati Taji Z, Bielytskyi P,** Shein M, Sani M-A, Seitz S, Schütz AK. Transient RNA Interactions Leave a Covalent Imprint on a Viral Capsid Protein. Journal of the American Chemical Society 2022;144:8536-8550.

[2] Porterfield JZ, Dhason MS, Loeb DD, Nassal M, Stray SJ, Zlotnick A. Full-length hepatitis B virus core protein packages viral and heterologous RNA with similarly high levels of cooperativity. J Virol 2010;84:7174-7184.

[3] Stevens TJ, Fogh RH, Boucher W, Higman VA, Eisenmenger F, Bardiaux B, et al. A software framework for analysing solid-state MAS NMR data. J Biomol NMR 2011;51:437-447.

[4] **Sacherl J, Kosinska AD**, Kemter K, Kächele M, Laumen SC, Kerth HA, et al. Efficient stabilization of therapeutic hepatitis B vaccine components by amino-acid formulation maintains its potential to break immune tolerance. JHEP Reports 2022.

[5] Porterfield JZ, Zlotnick A. A simple and general method for determining the protein and nucleic acid content of viruses by UV absorbance. Virology 2010;407:281-288.

[6] Cavaluzzi MJ, Borer PN. Revised UV extinction coefficients for nucleoside-5'-monophosphates and unpaired DNA and RNA. Nucleic Acids Res 2004;32:e13.

[7] Watkins NE, Jr., SantaLucia J, Jr. Nearest-neighbor thermodynamics of deoxyinosine pairs in DNA duplexes. Nucleic Acids Res 2005;33:6258-6267.

[8] Glasel JA. Validity of nucleic acid purities monitored by 260nm/280nm absorbance ratios. Biotechniques 1995;18:62-63.

[9] Trumpfheller C, Caskey M, Nchinda G, Longhi MP, Mizenina O, Huang Y, et al. The microbial mimic poly IC induces durable and protective CD4+ T cell immunity together with a dendritic cell targeted vaccine. Proc Natl Acad Sci U S A 2008;105:2574-2579.
