# Supplementary material for: Introducing adjuvant-loaded particulate hepatitis B core antigen as an alternative therapeutic hepatitis B vaccine component
Source: JHEP Rep. 2023 Dec 30;6(4):100997. doi: 10.1016/j.jhepr.2023.100997 (PMC10904195; doi:10.1016/j.jhepr.2023.100997)
Supplement: Multimedia component 2 [file mmc2.docx]

**Journal of Hepatology**

**CTAT methods**

Tables for a “Complete, Transparent, Accurate and Timely account” (CTAT) are now mandatory for all revised submissions. The aim is to enhance the reproducibility of methods.

- Only include the parts relevant to your study
- Refer to the CTAT in the main text as ‘Supplementary CTAT Table’
- Do not add subheadings
- Add as many rows as needed to include all information
- Only include one item per row

**If the CTAT form is not relevant to your study, please outline the reasons why:**

|  |
| --- |

- 1. **Antibodies**

| **Name** | **Citation** | **Supplier** | **Cat no.** | **Clone no.** |
| --- | --- | --- | --- | --- |
| Anti-mouse CD4 APC | Kosinska *et al*, Sci. Rep., 2019. | eBioscience | 17-0041-83 | GK1.5 |
| Anti-mouse CD8a PacificBlue | Kosinska *et al*, Sci. Rep., 2019. | BD Biosciences | 558106 | 53-6.7 |
| Anti-mouse IFNγ FITC | Kosinska *et al*, Sci. Rep., 2019. | BD Biosciences | 554411 | XMG1.2 |
| Anti-mouse TNFα PE-Cy7 | Michler and Kosinska *et al*, Gastroenterology, 2020. | BD Biosciences | 557644 | MP6-XT22 |
| anti-HBcoreAg 17H7 antibody |  | Helmholtz Monoclonal Antibody Core Facility |  |  |
| HRP-labeled secondary antibody |  | Helmholtz Monoclonal Antibody Core Facility |  |  |
| anti IFA HepBCore |  | Centro de Ingeniería Genética y Biotecnología de Cuba |  |  |
| HRP-labeled secondary antibody |  | Centro de Ingeniería Genética y Biotecnología de Cuba |  |  |
|  |  |  |  |  |
|  |  |  |  |  |
|  |  |  |  |  |

- 1. **Cell lines**

| **Name** | **Citation** | **Supplier** | **Cat no.** | **Passage no.** | **Authentication test method** |
| --- | --- | --- | --- | --- | --- |
| *E. coli* BL21(DE3) | Studier and Moffatt, J. Mol. Biol., 1986. | Novagen |  |  |  |

- 1. **Organisms**

| **Name** | **Citation** | **Supplier** | **Strain** | **Sex** | **Age** | **Overall n number** |
| --- | --- | --- | --- | --- | --- | --- |
| C57BL/6J | - | JANVIER LABS | C57BL/6 | male | 8-10 weeks | 120 |

- 1. **Sequence based reagents**

| **Name** | **Sequence** | **Supplier** |
| --- | --- | --- |
| B8R | TSYKFESV | peptides & elephants |
| C_93_ | MGLKFRQL | peptides & elephants |
| OVA_S8L_ | SIINFEKL | peptides & elephants |
| S_190_ | VWLSAIWM | peptides & elephants |
| S_208_ | IVSPFIPL | peptides & elephants |
| Cp2-188 | TWVGGNLEDPISRDLVVS | peptides & elephants |
| Cp2-189 | EDPISRDLVVSYVNTNMG | peptides & elephants |
| Cp2-190 | LVVSYVNTNMGLKFRQLL | peptides & elephants |
| Cp2-191 | TNMGLKFRQLLWFHISCL | peptides & elephants |
| Cp2-192 | RQLLWFHISCLTFGRETV | peptides & elephants |
| Cp2-193 | ISCLTFGRETVIEYLVSF | peptides & elephants |
| Cp2-194 | RETVIEYLVSFGVWIRTP | peptides & elephants |
| Cp2-195 | LVSFGVWIRTPPAYRPPN | peptides & elephants |
| Cp2-196 | IRTPPAYRPPNAPILSTL | peptides & elephants |
| Cp2-197 | RPPNAPILSTLPETTVVR | peptides & elephants |
| Cp2-198 | LSTLPETTVVRRRGRSPR | peptides & elephants |
| Sp3-37 | GNCTCIPIPSSWAFA | peptides & elephants |
| Sp3-38 | CIPIPSSWAFAKYLW | peptides & elephants |
| Sp3-39 | PSSWAFAKYLWEWAS | peptides & elephants |
| Sp3-40 | AFAKYLWEWASARFS | peptides & elephants |
| Sp3-41 | YLWEWASARFSWLSL | peptides & elephants |
| Sp3-42 | WASARFSWLSLLVPF | peptides & elephants |
| Sp3-43 | RFSWLSLLVPFVQWF | peptides & elephants |
| Sp3-44 | LSLLVPFVQWFVGLS | peptides & elephants |
| Sp3-45 | VPFVQWFVGLSPTVW | peptides & elephants |
| Sp3-46 | QWFVGLSPTVWLSAI | peptides & elephants |
| Sp3-47 | GLSPTVWLSAIWMMW | peptides & elephants |
| Sp3-48 | TVWLSAIWMMWYWGP | peptides & elephants |
| Sp3-49 | SAIWMMWYWGPSLYS | peptides & elephants |
| Sp3-50 | MMWYWGPSLYSIVSP | peptides & elephants |
| Sp3-51 | WGPSLYSIVSPFIPL | peptides & elephants |
| Sp3-52 | LYSIVSPFIPLLPIF | peptides & elephants |
| Sp3-53 | VSPFIPLLPIFFCLW | peptides & elephants |
| Sp3-54 | IPLLPIFFCLWVYI | peptides & elephants |

- 1. **Biological samples**

| **Description** | **Source** | **Identifier** |
| --- | --- | --- |
|  |  |  |

- 1. **Deposited data**

| **Name of repository** | **Identifier** | **Link** |
| --- | --- | --- |
|  |  |  |

- 1. **Software**

| **Software name** | **Manufacturer** | **Version** |
| --- | --- | --- |
| Flowjo | BD Bioscience | Version 10.7.2 |
| GraphPad Prism | GraphPad Software Inc | Version 5.01, 8.4.0 and 9.0.0 |
| CcpNmr Analysis | University of Leicester | Version 2 |
| TopSpin | Bruker | Version 3.5 |

- 1. **Other (e.g. drugs, proteins, vectors etc.)**

| **Protein Antigens** | **Supplier** |  |
| --- | --- | --- |
| HBsAg, genotype A | Biovac (Cape Town, South Africa) |  |
| HBcAg, genotype A | Center for Genetic Engineering and Biotechnology (Havana, Cuba) |  |
| HBcAg, genotype D | Dr. Dišlers, APP Latvijas Biomedicῑnas (Riga, Latvia) |  |
| **Viral vectors** |  |  |
| AAV-HBV 1.2 | Plateforme de Thérapie Génique in  Nantes, France (INSERM U1089) |  |
| MVA-S | Institute of Virology TUM/HMGU |  |
| MVA-core | Institute of Virology TUM/HMGU |  |

- 1. **Please provide the details of the corresponding methods author for the manuscript:**

| For the *in vitro* study, Zahra Harati Taji, [Zahra.Taji@cup.uni-muenchen.de](mailto:Zahra.Taji@cup.uni-muenchen.de), Ludwig Maximilians University of Munich / Technical University of Munich  For the *in vivo* study, Jinpeng Su, jinpeng.su@tum.de, Institute of Virology, Technical University of Munich / Helmholtz Munich. |
| --- |

**2.0 Please confirm for randomised controlled trials all versions of the clinical protocol are included in the submission. These will be published online as supplementary information.**

| Not relevant to our study. |
| --- |
